# Metabolic dysfunction-associated fatty liver disease in people living with HIV

**DOI:** 10.1038/s41598-023-32965-y

**Published:** 2023-06-06

**Authors:** Maurice Michel, Christian Labenz, Angelo Armandi, Leonard Kaps, Wolfgang Maximilian Kremer, Peter R. Galle, Daniel Grimm, Martin Sprinzl, Jörn M. Schattenberg

**Affiliations:** 1 Metabolic Liver Research Program, I. Department of Medicine, University Medical Centre Mainz, Mainz, Germany; 2 I. Department of Medicine, University Medical Centre Mainz, Mainz, Germany; 3 Department of Medical Sciences, University of Turin, Turin, Italy

**Keywords:** Hepatology, Liver diseases, Non-alcoholic fatty liver disease, HIV infections

## Abstract

The prevalence of metabolic risk factors and non-alcoholic fatty liver disease (NAFLD) is high among people living with HIV (PLWH). Data on the recently proposed definition of metabolic dysfunction-associated fatty liver disease (MAFLD) in PLWH receiving antiretroviral therapy (ART) remains unknown. A total of 282 PLWH were included in this cross-sectional cohort study. Vibration-controlled transient elastography (VCTE) was used to assess hepatic steatosis and fibrosis. MAFLD and its subgroups (overweight/obese, lean/normal weight, and type 2 diabetes) were defined according to a recently published international consensus statement. The majority of this cohort was male (n = 198, 70.2%), and the median age was 51.5 years. The median BMI was 25 kg/m^2^, and obesity was prevalent in 16.2% (n = 44). A total of 207 (73.4%) PLWH were classified as non-MAFLD while 75 (26.6%) qualified as MAFLD. The median CAP in the MAFLD group was 320 dB/m. PLWH with MAFLD showed a higher median LSM (p < 0.008) and were older (p < 0.005) compared to the non-MAFLD group. Overall, the metabolic risk profile was comparable between MAFLD and NAFLD. The majority of PLWH and MAFLD were overweight or obese (n = 58, 77.3%). The highest median LSM values were observed in the subgroup with MAFLD and type 2 diabetes. HIV-related parameters did not differ between non-MAFLD and MAFLD. The prevalence of MAFLD in PLWH is high and comparable to NAFLD. PLWH may be characterized according to the novel MAFLD criteria and its subgroups to identify patients at risk for chronic liver disease.

## Introduction

The prevalence of non-alcoholic fatty liver disease (NAFLD) is estimated at 25% globally^1^. Abnormal liver function tests can be observed in up to a fifth of the general population with 1.1% exhibiting advanced fibrosis^2^. The metabolic syndrome and its associated risk factors have become a key driver for the development and progression of NAFLD and its inflammatory subtype non-alcoholic steatohepatitis (NASH)^3^. Hepatic inflammation leads to progressive scarring of liver tissue and the stage of liver fibrosis has been linked to hepatic- and extrahepatic morbidity and mortality^4,5^. Previous studies have shown a high prevalence of NAFLD in people living with HIV (PLWH) as a result of an aging population, a high prevalence of metabolic risk factors, and HIV-related parameters^6–9^. Besides external nutritional factors, it has been proposed that antiretroviral therapy (ART) imposes negative metabolic side effects leading to weight gain and hepatic steatosis^10,11^. Moreover, metabolic comorbidities and hepatic steatosis show a negative impact on the health-related quality of life in PLWH^12^.

Recently, the term metabolic dysfunction-associated fatty liver disease (MAFLD) has been proposed to provide positive criteria, reduce stigmatization and avoid histological definitions of liver disease^13^. The ongoing academic discussion around this terminology has many facets, including regulatory aspects of a name change but also public health aspects including ICD-10 coding. MAFLD is defined by the presence of hepatic steatosis using an imaging modality and metabolic risk factors regardless of alcohol intake or other causes of liver disease. Since alcohol consumption is presumably often under or overreported, MAFLD may overcome these limitations. This may also be important in PLWH, as several pro-steatogenic factors, including HIV infection, ART, and viral hepatitis, are more prevalent^8,11,14^. Additionally, MAFLD specifically identifies lean and normal-weight individuals when additional risk factors are present.

In clinical practice, non-invasive tests (NITs) are used to stage liver disease in patients in the absence of liver histology^15^. Vibration-controlled transient elastography (VCTE) can be used for point-of-care screening of hepatic steatosis and fibrosis^16^. Only few data on the prevalence of hepatic steatosis and fibrosis according to the recently developed definition of MAFLD in PLWH are available, and importantly these studies have been predominantly conducted in Asia^17–19^. The aim of this analysis was to compare the clinical and liver-specific characteristics of PLWH using both definitions of MAFLD and NAFLD and to explore if subcategories within the broader spectrum of MAFLD exhibit more advanced liver disease or comorbidities.

## Methods

### Study population

**Figure 1:**
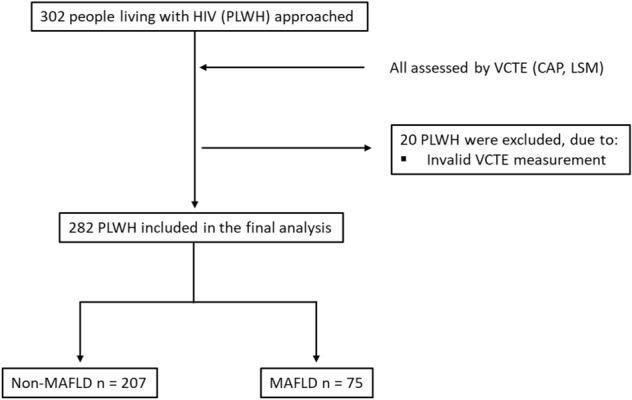
Flow diagram showing the exclusion of ineligible participants. Vibration controlled transient elastography (VCTE), controlled attenuation parameter (CAP), liver stiffness measurement (LSM).

A total of 302 PLWH have been approached for this monocentric cohort study (FLASH, Prevalence of Advanced Fibrosis in Patients Living With HIV) at the outpatient clinic of the Metabolic Liver Research Program at the University Medical Centre Mainz in Germany. Of these individuals, 282 were included in the final analysis. Participants had to be at least 18 years of age and provide written informed consent before study inclusion. If participants had an active malignancy, they were excluded from the study. Data were collected at baseline and accessible through the medical health care records. A study flow diagram is shown in Fig. 1.

### Non-invasive assessment of hepatic steatosis and fibrosis

Vibration-controlled transient elastography (VCTE, FibroScan^®^ 430 mini; SMART Exam was introduced in 2020; Echosens, Paris, France) was used to non-invasively assess hepatic steatosis (CAP, dB/m) and fibrosis (LSM, kPa)^16^. The M probe was used in 91.1%, and the XL probe in 8.9%. A total of 20 participants (7%) were excluded due to invalid VCTE scans, as previously described^9^. Hepatic steatosis was defined with a cut-off value of ≥ 275 dB/m (CAP) according to the recently published EASL guidelines on NITs^20^. A cut-off value of ≥ 8.2 kPa (LSM) was considered significant fibrosis (≥ F2)^21^.

Additional surrogate scores of hepatic steatosis and fibrosis included the fatty liver index (FLI) to categorize the likelihood of hepatic steatosis^22^, the NAFLD fibrosis score (NFS), AST to Platelet Ratio Index (APRI) and Fibrosis-4 (FIB-4) score using published cut-off values to rule in or rule out fibrosis^23–25^. The Fibroscan-AST score (FAST score), which includes LSM, CAP, and AST to rule out (lower cut-off < 0.35) or rule in (upper-cut-off > 0.67) steatohepatitis with significant fibrosis was used^26^.

### Definition of MAFLD and NAFLD

Metabolic dysfunction-associated fatty liver disease (MAFLD) was defined according to a recently published international consensus statement^13^. For a diagnosis of MAFLD, hepatic steatosis, defined by CAP ≥ 275 dB/m, had to be present. In addition to hepatic steatosis, one of the following three criteria had to be evident: overweight/obesity (≥ 25 kg/m^2^), lean/normal weight (< 25 kg/m^2^) with evidence of metabolic risk factors or type 2 diabetes mellitus (T2DM). At least two of the following metabolic risk abnormalities had to be present in the lean/normal weight group: waist circumference ≥ 102/88 cm in men and women, respectively, blood pressure ≥ 130/85 mmHg or specific drug treatment, plasma triglyceride (TG) levels ≥ 150 mg/dl or specific drug treatment, plasma HDL-cholesterol < 40 mg/dl for men and < 50 mg/dl for women or specific drug treatment or prediabetes (fasting glucose 100–125 mg/dl or HbA1c 5.7–6.4%)^13^. MAFLD-only included participants that fulfilled the criteria of MAFLD but not NAFLD. Thus, this subgroup also included participants with an alcohol intake exceeding > 20 g/day in males and > 10 g/day in females.

Non-alcoholic fatty liver disease (NAFLD) was defined according to current European practice guidelines^27^. Cut-offs for alcohol consumption (g/day) were assessed clinically and defined as no more than 20 g/day for males and 10 g/day for females. NAFLD-only included participants that fulfilled the criteria of a NAFLD but not a MAFLD. Overlap MAFLD/NAFLD refers to PLWH that fulfilled the definition of both entities with a CAP ≥ 275 dB/m and an alcohol intake of less than 20 g/day (males) and 10 g/day (females).

### Definition of demographic variables

Body mass index (BMI, kg/m^2^; weight (kg)/height^2^ (m^2^)) and waist circumference (cm) were assessed at the time of enrollment. A higher education was considered for participants that had at least a high school diploma or above (college degree), whereas a lower education was considered for patients with any degree below a high school diploma. Comorbidities were retrieved from the patient’s history or medical records. Laboratory values were assessed at baseline at the time of enrollment.

### Statistics

Descriptive analysis of data is expressed as median values with interquartile ranges (IQR 25th; 75th). The Mann–Whitney *U* rank test and the Kruskal Wallis test were used to compare groups and to calculate differences between two groups or more with continuous variables. Categorical variables are presented as frequencies and percentages. The chi-square test was used to compare two or more patient groups. All tests were two-tailed; statistically significant values were defined as p < 0.05. IBM SPSS Statistic Version 23.0 (Armonk, NY: IBM Corp.) was used for all data analyses and statistical tests. Either Microsoft Excel 2016 or Microsoft PowerPoint 2016 (Redmond, WA: Microsoft Corp.) was used for all figures.

### Ethical approval

The study was approved by the ethics committee of the Landesärztekammer Rhineland-Palatine (Nr. 873.199.10 (7208)). The study was conducted according to the ethical guidelines of the 1975 Declaration of Helsinki (6th revision, 2008).

### Informed consent

Informed consent was obtained from all participants involved in the study.

## Results

### Baseline characteristics and a comparison of PLWH and non-MAFLD vs. MAFLD

The baseline characteristics of non-MAFLD compared to MAFLD are summarized in Table 1. A total of 207 (73.4%) PLWH were classified as non-MAFLD and 75 (26.6%) as MAFLD. Applying a lower CAP cut-off of ≥ 248 dB/m, the prevalence of MAFLD was 39.4% (n = 111). Individuals with MAFLD were older (p = 0.005) compared to non-MAFLD. Sleep apnea syndrome was more prevalent in MAFLD (p = 0.013). The median CAP was 320 dB/m (IQR 293; 343) in PLWH and MAFLD, compared to the median CAP of 233 dB/m (IQR 207; 258) in non-MAFLD. The median LSM was higher in PLWH and MAFLD, whereas no difference was observed in the number of participants exhibiting significant fibrosis. In line with the definition of MAFLD, metabolic comorbidities were particularly prominent in PLWH and MAFLD. Median levels of ALT (U/I) and GGT (U/l) were higher in MAFLD compared to non-MAFLD (p = 0.001; p < 0.001). When comparing MAFLD with NAFLD, no major differences were observed. Importantly, the metabolic risk profiles were comparable in PLWH with MAFLD and NAFLD. An in-depth analysis of PLWH and NAFLD has been previously described^9^.

**Table 1:** Baseline characteristics of study population and comparison between PLWH and non-MAFLD vs. MAFLD.

	Non-MAFLD	MAFLD	NAFLD	P
N	207	75	76	
Variables	n (% or IQR)	n (% or IQR)	n (% or IQR)	Non-MAFLD vs. MAFLD
Age (years)	50 (41; 57)	55 (47; 60)	54 (49; 60)	**0.005**
Time since diagnosis (years) n = 268	12 (6; 19)	13 (5; 23)	14 (6; 23)	0.780
Sex				0.201
Male	141 (68.1)	57 (76)	60 (78.9)	
Female	66 (31.9)	18 (24)	16 (21.1)	
Education n = 252				0.213
Higher	56 (30.7)	16 (22.8)	16 (22.9)	
Lower	126 (69.2)	54 (77.2)	54 (77.1)	
Unemployed n = 252	13 (7.1)	10 (14.3)	10 (14.3)	0.078
Comorbidities				
Sleep apnea syndrome n = 197	28 (18.6)	20 (35.1)	16 (29.6)	**0.013**
Hypothyroidism n = 197	8 (5.8)	4 (6.9)	2 (3.7)	0.760
Myocardial infarction n = 194	11 (7.9)	4 (7.4)	4 (8)	0.916
Stroke n = 194	5 (3.6)	5 (9.1)	5 (9.8)	0.119
VCTE				
CAP	233 (207; 258)	320 (293; 343)	314 (293; 343.8)	**< 0.001**
LSM	4.5 (3.7; 5.5)	5 (4.1; 6.2)	5 (4.1; 6.2)	**0.008**
≥ 8.2	12 (5.8)	7 (9.3)	7 (9.2)	0.295
Metabolic comorbidities				
BMI (kg/m^2^) n = 272	23.4 (21.7; 26.3)	28.7 (26.8; 33.5)	27.7 (25.4; 31.6)	**< 0.001**
Obesity (> 30 kg/m^2^) n = 272	14 (7.1)	30 (40)	26 (35.1)	**< 0.001**
Waist circumference (cm) n = 270	92 (84; 100)	106 (98; 115.3)	102 (97; 113)	**< 0.001**
Male > 102 cm	25 (18.8)	35 (62.5)	28 (49.1)	**< 0.001**
Female > 88 cm	31 (49.2)	18 (100)	16 (100)	**< 0.001**
Type 2 diabetes n = 261	17 (8.6)	13 (20.3)	11 (16.9)	**0.011**
High triglycerides n = 175	39 (31.7)	32 (61.5)	33 (61.1)	**< 0.001**
High cholesterol n = 181	56 (43.4)	35 (67.3)	33 (61.1)	**0.004**
Arterial Hypertension n = 268	53 (26.6)	32 (46.4)	27 (39.1)	**0.002**
High alcohol intake	15 (8.2)	10 (13.3)	0	0.205
Laboratory values				
ALT (U/l) n = 263	22.5 (17; 30)	28 (20; 40)	28 (18.3; 38)	**0.001**
AST (U/l) n = 263	26 (22; 30.7)	26 (23; 34)	26 (23; 32.8)	0.600
GGT (U/l) n = 258	26 (18; 39)	35 (24; 60)	31 (22.3; 52)	**< 0.001**
Triglycerides (mg/dl) n = 175	111 (85; 173)	180.5 (125.3; 234.5)	183 (123.8; 246.3)	**< 0.001**
Cholesterol (mg/dl) n = 181	195 (172; 220)	212 (184; 241.3)	207 (182.8; 228)	**0.012**
HDL (mg/dl) n = 133	48 (40; 59)	46.5 (38; 55.3)	43.5 (38; 52)	0.383
LDL (mg/dl) n = 133	118 (102; 143)	126.5 (114; 150.5)	122.5 (103.5; 143.5)	0.169
HbA1c (%) n = 136	5.4 (5.1; 5.7)	5.5 (5.2; 6.1)	5.5 (5.3; 6.1)	0.223
Uric acid (mg/dl) n = 147	5.3 (4.6; 6.1)	6.2 (5.5; 7)	6 (5.1; 6.9)	**0.001**
Albumin (g/l) n = 157	40 (38; 42)	40 (39; 42)	41 (39; 46)	0.556
Creatinine (mg/dl) n = 263	0.92 (0.79; 1.1)	0.98 (0.82; 1)	0.98 (0.84; 1.1)	0.235
Platelets (/nl) n = 277	234 (196; 273)	244 (206; 276.5)	239 (199; 278)	0.187
Leukocytes (/nl) n = 277	6.5 (5.2; 7.8)	6.7 (5.5; 7.7)	6.9 (5.8; 7.8)	0.440
Non-invasive tests				
FLI n = 162	30 (14; 55.3)	76.5 (66; 94.8)	72 (50.5; 91.8)	**< 0.001**
FLI > 60	23 (20.9)	43 (82.7)	38 (73.1)	**< 0.001**
APRI n = 263	0.3 (0.2; 0.4)	0.3 (0.2; 0.4)	0.3 (0.3; 0.4)	0.874
APRI > 1.5	4 (2.1)	2 (2.8)	2 (2.7)	0.311
NFS n = 152	− 2.085 (− 2.897; − 1.270)	− 1.320 (− 2.370; − 0.752)	− 1.465 (− 2.485; − 0.890)	**0.005**
NFS > − 1.455	30 (27.8)	23 (52.3)	23 (50)	**0.004**
FIB-4 n = 262	1.1 (0.8; 1.5)	1.1 (0.9; 1.4)	1.1 (0.9; 1.5)	0.638
FIB-4 > 1.3	74 (38.7)	23 (32.4)	24 (33.3)	0.344
FAST score n = 263	0.10 (0.07; 0.18)	0.17 (0.13; 0.35)	0.17 (0.13; 0.35)	**< 0.001**
FAST score > 0.35	14 (7.3)	18 (25.4)	18 (25)	**< 0.001**
FAST score > 0.67	5 (2.6)	3 (4.2)	2 (2.8)	0.497

Data are expressed as numbers, median, percentage (%) or interquartile ranges (IQR 25th; 75th). p values refer to the comparison between non-MAFLD vs. MAFLD. Boldface indicates statistical significance. A p value < 0.05 was considered statistically significant.

Next, we explored commonly used NITs to detect steatosis and fibrosis in PLWH and MAFLD. With a cutoff of > 60 using the FLI, more PLWH were identified in the MAFLD group (82.7%, p < 0.001) than in the non-MAFLD group. The NFS detected more PLWH with significant fibrosis in the MAFLD group (p = 0.004), although no difference was seen using the FIB-4 and the APRI score (p = 0.344; p = 0.311). The FAST score ruled in 25.4% of PLWH and MAFLD, suggesting a high prevalence of NASH with significant fibrosis in this group.

The use of tenofovir alafenamide (TAF) and integrase inhibitors (INSTI) as part of the ART regimen was numerically higher in PLWH and MAFLD, and fewer individuals used TDF compared with non-MAFLD. Overall, no difference was seen in the comparison of HIV-related parameters or ART between non-MAFLD vs. MAFLD (Supplementary Table 1).

### Comparison of PLWH across the spectrum of NAFLD and MAFLD

**Figure 2:**
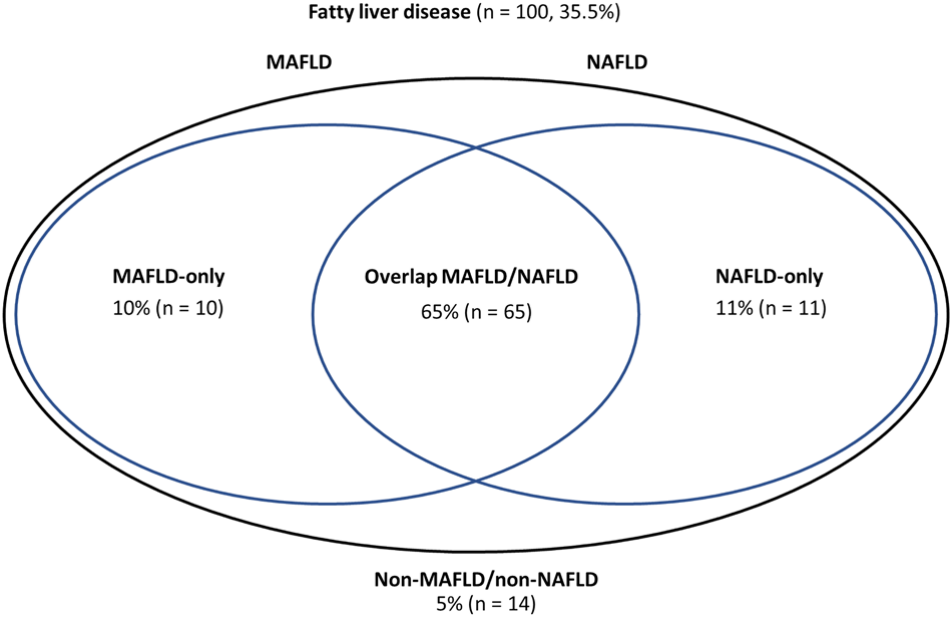
The Venn diagram showing the distribution of participants with MAFLD-only, overlap MAFLD/NAFLD, NAFLD-only and non-MAFLD/non-NAFLD.

Next, we compared MAFLD and NAFLD to identify potential overlaps and differences in PLWH (Fig. 2). The comparison of these subgroups (overlap MAFLD/NAFLD, MAFLD-only, and NAFLD-only) is summarized in Table 2. The median LSM (kPa) values were similar among all subgroups. The group of PLWH and overlap MAFLD/NAFLD exhibited the highest prevalence of significant fibrosis. Individuals with NAFLD-only were younger and showed a lower metabolic risk profile compared to the other subgroups. The median BMI (kg/m^2^) and waist circumference (cm) were lower in NAFLD-only (p < 0.001). Liver enzymes were higher in individuals with MAFLD-only, and the median levels of GGT (U/l) were significantly elevated in this subgroup. The FLI showed the lowest scores in NAFLD-only (p < 0.001) and the highest score was seen in MAFLD-only (p = 0.003). Concordantly, a cutoff > 60 using the FLI identified more individuals in the overlap MAFLD/NAFLD and MAFLD-only groups, respectively. The FIB-4 identified more individuals in both, MAFLD-only and NAFLD-only, subgroups. Study participants within the non-MAFLD/non-NAFLD subgroup either had high alcohol consumption or lacked information on alcohol consumption, hence no classification was possible (data not shown).

**Table 2:** Comparison of clinical parameters between overlap MAFLD/NAFLD and MAFLD-only or NAFLD-only.

Variables	Overlap MAFLD/NAFLD	MAFLD-only	NAFLD-only	P		
N	65	10	11	Overlap vs. MAFLD-only	Overlap vs. NAFLD-only	MAFLD-only vs. NAFLD-only
Age (years)	55 (49; 61)	54 (40; 58.5)	50 (42; 55)	0.454	0.172	0.549
Time since diagnosis (years)	14 (6; 23)	6 (3.5; 16)	14.5 (5.3; 19.3)	0.108	0.546	0.426
Sex				0.750	0.064	0.119
Male	49 (75.4)	8 (80)	11(100)			
Female	16 (24.6)	2 (20)	0			
Education				**0.027**	**0.027**	1
Higher	11 (18.3)	5 (50)	5 (50)			
Lower	49 (81.6)	5 (50)	5 (50)			
Unemployed	10 (16.7)	0	0	0.163	0.163	0
Comorbidities						
Sleep apnea syndrome	15 (30.9)	5 (50)	1 (14.3)	0.277	0.341	0.129
Hypothyroidism	2 (4.2)	2 (20)	0	0.072	0.610	0.242
Myocardial infarction	4 (9)	0	0	0.322	0.441	0
Stroke	5 (11.1)	0	0	0.269	0.390	0
VCTE						
CAP	320 (294.5; 343.5)	318 (287.8; 346.3)	297 (278; 361)	0.749	0.255	0.468
LSM	5 (4.2; 6.3)	4.7 (3.9; 5.9)	4.5 (3.7; 5.3)	0.502	0.240	0.778
≥ 8.2 kPa	7 (10.8)	0	0	0.276	0.253	0
Metabolic comorbidities						
BMI (kg/m^2^)	29 (26.2; 32.7)	28.6 (28.3; 33.9)	22.4 (21.3; 23.5)	0.418	**< 0.001**	**< 0.001**
Waist circumference (cm)	104.5 (98; 114.8)	110 (105.5; 119.5)	89 (86.5; 96)	0.192	**< 0.001**	**0.001**
Type 2 diabetes	11 (20.4)	2 (20)	0	0.979	0.101	0.119
High triglycerides	29 (64.4)	3 (42.9)	4 (44.4)	0.275	0.261	0.949
High cholesterol	30 (66.7)	5 (71.4)	3 (33.3)	0.803	0.061	0.131
Arterial hypertension	26 (44.1)	6 (60)	1 (10)	0.350	**0.041**	**0.019**
Laboratory values						
ALT (U/l)	28 (19.5; 40)	30.5 (23.8; 47)	26 (16; 36)	0.519	0.429	0.306
AST (U/l)	25 (23; 34)	32 (24.5; 38.8)	28 (24; 30)	0.136	0.442	0.377
GGT (U/l)	31 (22.5; 55)	79.5 (35.5; 131)	29 (19; 40)	**0.005**	0.298	**0.008**
Triglycerides (mg/dl)	185 (132; 241.5)	125 (83; 183)	141 (79; 290)	0.072	0.634	0.491
Cholesterol (mg/dl)	212 (185; 232)	239 (173; 288)	196 (155; 210.5)	0.348	0.123	0.112
HDL (mg/dl)	41 (38; 51.8)	69.5 (61.8; 76.5)	48 (38; 61.5)	**0.004**	0.483	**0.014**
LDL (mg/dl)	126.5 (114.3; 149.5)	127.5 (81.3; 173)	107.5 (88.5; 132.5)	0.999	0.097	0.439
HbA1c (%)	5.5 (5.1; 6.1)	5.5 (5.2; 6.7)	5.5 (5.4; 5.8)	0.706	0.734	0.949
Uric acid (mg/dl)	6.2 (5.4; 7.1)	5.9 (5.5; 7)	5.2 (4.5; 5.9)	0.894	0.080	0.139
Albumin (g/l)	40.5 (39; 42)	39 (34.3; 41.5)	42 (40; 43.5)	0.174	0.300	0.085
Creatinine (mg/dl)	0.99 (0.84; 1.1)	0.91 (0.77; 0.98)	0.93 (0.82; 1.1)	0.133	0.524	0.526
Platelets (/nl)	241 (207.5; 278.8)	247 (188.3; 258)	200 (181; 225)	0.492	0.053	0.324
Leukocytes (/nl)	6.9 (5.8; 7.8)	5.7 (4.7; 7.1)	6.7 (5.1; 7.3)	0.079	0.441	0.324
Non-invasive tests						
FLI	74 (61; 94)	93 (86; 95)	28 (18; 61)	0.129	**< 0.001**	**0.003**
FLI > 60	36 (80)	7 (100)	2 (28.6)	0.193	**0.004**	**0.005**
APRI	0.3 (0.2; 0.5)	0.4 (0.3; 0.6)	0.4 (0.3; 0.5)	0.098	0.127	0.888
APRI > 1.5	2 (3.3)	0	0	0.561	0.542	0
NFS	− 1.320 (− 2.400; − 0.857)	− 1.140 (− 2.397; 0.157)	− 1.700 (− 3.565; − 1.215)	0.473	0.235	0.197
NFS > − 1.455	20 (52.6)	3 (50)	3 (37.5)	0.905	0.437	0.640
FIB-4	1.1 (0.9; 1.4)	1.4 (0.7; 1.9)	1.6 (0.9; 1.8)	0.482	0.112	0.751
FIB-4 > 1.3	17 (27.8)	6 (60)	7 (63.6)	**0.044**	**0.021**	0.864
FAST score	0.16 (0.13; 0.35)	0.23 (0.12; 0.38)	0.19 (0.12; 0.33)	0.869	0.820	0.573
FAST score > 0.35	16 (26.2)	2 (20)	2 (18.2)	0.675	0.570	0.916
FAST score > 0.67	2 (3.3)	1 (10)	0	0.327	0.542	0.283

Data are expressed as numbers, median, percentage (%) or interquartile ranges (IQR 25th; 75th). Boldface indicates statistical significance. A p value < 0.05 was considered statistically significant.

The comparison with HIV-related parameters showed no significant differences (Supplementary Table 2). A higher number of individuals received TAF and INSTI, although the numbers were equally distributed between the groups. A numerically lower number of PLWH received INSTI in the NAFLD-only group.

### Prevalence and differences in MAFLD subgroups

**Figure 3:**
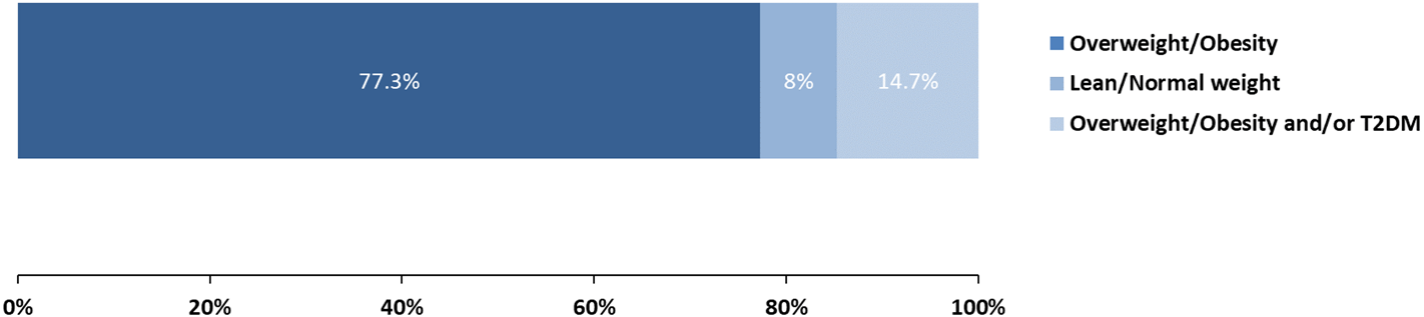
Prevalence of each subgroup of MAFLD.

The prevalence and characteristics of the different subgroups of MAFLD according to metabolic risk profile, NITs, and HIV-related parameters are shown in Fig. 3 and Table 3. The majority of PLWH and MAFLD were within the overweight/obesity group (n = 58, 77.3%). The second largest group consisted of individuals with overweight/obesity and/or T2DM (n = 11; 14.7%), and 6.8% of PLWH were categorized in the lean/normal weight group (n = 6; 8%).

**Table 3:** Comparison of MAFLD subgroups according to metabolic risk profile, non-invasive tests and HIV-related parameters.

Variables	Overweight/obesity	Lean/normal weight	Overweight/obesity and/or T2DM	p
N	58	6	11	
Metabolic risk abnormalities				
Waist circumference	108 (99.5; 116.5)	97 (94.3; 98)	104 (96; 113)	**0.006**
Hypertension	23 (44.2)	4 (66.7)	5 (45.5)	0.579
Triglycerides	174.5 (108.8; 230.5)	135 (116; 158)	233 (187; 314)	**0.018**
High TG	22 (57.9)	1 (20)	9 (100)	**0.009**
HDL	46.5 (38; 57.3)	40 (38; 54.4)	47 (35; 52)	0.618
Low HDL	13 (40.6)	2 (66.7)	1 (14.3)	0.254
HbA1c	5.4 (5.1; 5.9)	5.3 (4.8; 5.9)	6.6 (6.2; 7.8)	**0.010**
Prediabetes	9 (31)	2 (50)		0.675
High alcohol intake	10 (17.3)	0	0	0.184
Non-invasive tests				
CAP (dB/m)	317 (292.3; 345.3)	323 (295; 331.3)	315 (305; 352)	0.870
LSM (kPa)	5 (4; 6.2)	4.7 (3.8; 5.1)	6.3 (5; 7.6)	**0.038**
≥ 8.2 kPa	5 (8.6)	0	2 (18.2)	0.434
FLI	85 (67.5; 95.5)	38 (26; 44)	80 (74.5; 87.5)	**0.002**
FLI > 60	34 (89.5)	0	9 (90)	** < 0.001**
APRI	0.34 (0.25; 0.48)	0.25 (0.18; 0.28)	0.28 (0.24; 0.47)	**0.038**
APRI > 1.5	2 (3.6)	0	0	0.741
FIB-4	1.1 (0.9; 1.5)	0.9 (0.8; 1.3)	1.1 (0.9; 1.2)	0.520
FIB-4 > 1.3	21 (38.2)	1 (16.7)	1 (10)	0.149
NFS	− 0.138 (− 2.427; − 0.322)	− 2.470 (− 3.332; − 2.350)	− 1.125 (− 1.360; − 0.895)	**0.048**
NFS > − 0.45	16 (50)	0	7 (87.5)	**0.015**
FAST-score	0.201 (0.130; 0.350)	0.137 (0.101; 0.188)	0.254 (0.149; 0.397)	0.218
> 0.35	14 (25.5)	0	4 (40)	0.205
> 0.67	3 (5.5)	0	0	0.634
HIV-related parameters				
RNA viral load				**0.012**
< 50 copies/ml	41 (71.9)	2 (33.3)	3 (30)	
> 50 copies/ml	16 (28.1)	4 (66.7)	7 (70)	
CD4 (cells/µl)	773 (533.5; 1019.3)	828.5 (590.3; 1269.5)	823 (292.3; 985)	0.865
> 500 CD4 cells/µl	43 (74.1)	5 (83.3)	7 (63.6)	0.652
CDC stages				
C	10 (27.8)	2 (33.3)	2 (25)	0.727
NRTI (TAF vs. TDF)				0.830
TAF	40 (90.9)	5 (83.3)	7 (87.5)	
TDF	4 (9.1)	1 (16.6)	1 (12.5)	
INSTI	43 (76.8)	4 (66.7)	7 (63.6)	0.604
PI	7 (12.5)	1 (16.7)	2 (18.2)	0.861
NRRTI	11 (19.6)	1 (16.7)	3 (27.3)	0.824

Data are expressed as numbers, median, percentage (%) or interquartile ranges (IQR 25th; 75th). p values refer to the comparison between all subgroups of MAFLD for which the Kruskal Wallis test was used. Boldface indicates statistical significance. A p value < 0.05 was considered statistically significant.

The median CAP was numerically higher in lean/normal-weight individuals. PLWH in the T2DM group had the highest median LSM value. Exploration of the surrogate scores showed that the FLI failed to identify hepatic steatosis in the lean/normal weight group, but not in the other two subgroups. Likewise, the FIB-4 identified more individuals in the overweight/obesity group with an LSM ≥ 8.2 kPa, while the NFS detected more individuals in the T2DM subgroup.

Overall, no significant differences in ART were seen among these subgroups, although the majority of PLWH were treated with TAF and INSTI in all subgroups. A comparison of HIV-related parameters among these subgroups is shown in Table 3.

## Discussion

In this study, we analyzed the recently proposed definition of MAFLD in a German cohort of PLWH. As expected, we observed a large overlap between the definitions of MAFLD and NAFLD. The higher prevalence of metabolic comorbidities in PLWH and MAFLD relates to the inclusion of these in the disease definition. Whereas the groups—overlap MAFLD-NAFLD and MAFLD-only—were largely comparable to each other, the NAFLD-only group had a lower prevalence of metabolic risk factors and significant liver fibrosis. According to the proposed subgroups of MAFLD, PLWH presenting with overweight/obesity and T2DM show a higher risk profile to develop fibrosis. In turn, HIV-related parameters were not different between MAFLD and non-MAFLD.

In previous analyses, PLWH showed a higher prevalence of fatty liver disease in comparison to HIV-negative individuals^7^. The prevalence of MAFLD was 26.6% in this cohort. Other studies analyzing MAFLD in cohorts from Asia reported a higher prevalence, including 35% in China and 32% in Thailand. Importantly, these studies examined a lower cut-off of 248 dB/m and thus likely overestimated the prevalence of MAFLD^17,18^. Applying the cut-off of 248 dB/m, the prevalence of MAFLD increased to 39.4% in this cohort. A study from Germany reported hepatic steatosis in 48.5% of PLWH using a lower cut-off at 238 dB/m^10^. While studies in PLWH suggest the use of 248 dB/m as a cut-off for hepatic steatosis, current practice guidelines on NITs recommend to use of a cut-off of 275 dB/m regardless of HIV status^20,28^. The reported prevalence is affected by the chosen cut-off, and the optimal cut-off in PLWH remains to be determined.

Several factors, including HIV infection and ART, have been proposed to have pro-steatogenic and negative metabolic effects^29^. An important observation in this cohort study of well-controlled PLWH, all of whom have access to a publicly funded health care system, was that HIV-defining variables did not differ across the cohort studied. Recent studies have highlighted the impact of certain ART regimens on weight gain and an increase in hepatic steatosis in PLWH^10,11^. The use of tenofovir alafenamide (TAF) has been discussed in the context of emergent obesity and worsening of serum lipid levels compared to tenofovir disoproxil fumarate (TDF)^11^. Only recently, Bischoff et al. showed the impact of TAF and integrase inhibitors (INSTI) instead of TDF on steatosis progression^10^. However, TDF is increasingly replaced by TAF in Germany related to a better safety profile^30,31^. Cumulative exposure to INSTI remained an independent predictor to develop MAFLD in a cohort from China^18^. In fact, the majority of our cohort received treatment with TAF and INSTI, but no differences were seen between non-MAFLD and MAFLD. In addition, the cohorts from Asia were considerably younger compared to PLWH in our study^17,18^. In comparison to a recent study that explored the prevalence of hepatic steatosis using the FLI in the general population, participants with MAFLD in the current analysis were also younger^2^. Therefore, PLWH may develop hepatic steatosis at a younger age, which is in part a consequence of HIV infection. However, we were not able to detect an impact of HIV-related parameters in this analysis, and factors unrelated to viral replication, e.g. social status, income, or education could contribute to emergent hepatic steatosis.

The MAFLD definition in this study also compromised participants with an alcohol intake of more than 20 g/day (males) and 10 g/day (females). Alcohol consumption was unknown in a smaller number of participants (3.9%), although they would have met MAFLD criteria for metabolic risk factors. Applying the criteria of MAFLD may overcome this issue if alcohol consumption remains unknown. Previous studies have detected more severe liver injury with higher rates of hepatic fibrosis in patients with MAFLD and alcohol consumption^32,33^. Participants with MAFLD-only represented those with a higher alcohol intake in our study. This subgroup showed elevated ALT, AST, and GGT levels but lower LSM results compared to PLWH with a lower alcohol intake. Nevertheless, higher alcohol intake may be a co-risk factor in addition to dysmetabolism in MAFLD^34^. In the NAFLD-only group, a lower age, normal weight, and lower frequency of metabolic risk factors were present. This subgroup with lean NAFLD in the absence of metabolic comorbidities is currently overlooked in the MAFLD definition^13^. Notably, overlap MAFLD/NAFLD had a high prevalence of metabolic risk factors with higher numbers of T2DM, liver fibrosis, and other extrahepatic comorbidities. This is in line with previous studies of HIV-negative MAFLD patients^33^.

The analysis of MAFLD subgroups revealed an increased prevalence of overweight and obesity. Besides the BMI, the median waist circumference was the highest in MAFLD. In this context, a previous analysis has shown that an android fat distribution is also an important factor in disease progression, especially in females^35^. However, the more severely affected subgroup included PLWH and T2DM, of whom the majority were also overweight or obese. Both comorbidities are known risk factors for disease progression and worsening hepatic fibrosis with a higher risk of developing hepatocellular carcinoma (HCC)^36–38^. The median LSM was the highest in this subgroup with T2DM. A recent meta-analysis showed that elevated LSM findings were present in almost 20% of patients with T2DM^39^. Surprisingly, PLWH in the subgroup with normal weight showed the highest median CAP values compared to the other subgroups. In addition, the median FLI was low and a cut-off > 60 did not reveal positive findings in this subgroup. Thus, VCTE appears to be superior to other NITs, especially in patients with normal weight and fewer metabolic comorbidities. In the subgroups of PLWH being overweight and having T2DM, the NFS detected a higher number of individuals with LSM ≥ 8.2 kPa. Considering the accuracy and overall availabilities of NITs other than VCTE, their role in PLWH may be comparable to the general population for screening for liver disease. Future studies are needed to validate these fibrosis scores in patients with MAFLD, including HIV-positive individuals.

Limitations of this study include the inaccuracy of NITs to detect hepatic steatosis and fibrosis. The used cut-offs to define significant fibrosis and hepatic steatosis are determining the overall results and observed associations. Despite the advantages of the MAFLD definition, the inclusion of high alcohol intake may be a potential confounder. Furthermore, because of the lack of a longitudinal design, we are unable to provide data on cumulative exposure to specific ART regimens that could potentially affect the development of hepatic steatosis over time. Moreover, information on previous exposure to ART is missing. Nevertheless, we present data from a large and well-characterized cohort of PLWH that have been screened non-invasively for hepatic steatosis and fibrosis by VCTE.

The prevalence of MAFLD was high in this cohort and was related to the high prevalence of metabolic risk factors in PLWH. Characterizing PLWH according to subgroups of MAFLD may be useful to identify those patients at particular risk to develop advanced liver disease. Overall, MAFLD and NAFLD showed similar aspects, especially in terms of hepatic fibrosis. Therefore, future longitudinal analyses are needed to compare the individual impact of both definitions, MAFLD and NAFLD, and the impact of HIV-related parameters on hepatic- and extrahepatic morbidity in PLWH.

## Supplementary Information

Supplementary Information.

## Data Availability

The data presented in this study are available on request from the corresponding author.

## Abbreviations

ALT: Alanine transaminase
APRI: AST-to-platelet ratio index
ART: Antiretroviral therapy
AST: Aspartate transaminase
BMI: Body mass index
BIC: Bictegravir
CAP: Controlled attenuation parameter
CDC: Centers for disease control and prevention
DTG: Dolutegravir
EVG: Elvitegravir
FAST score: Fibroscan-AST score
FIB-4: Fibrosis-4 index
FLI: Fatty liver index
GGT: Gamma-glutamyl transferase
HDL: High-density lipoprotein
INSTI: Integrase inhibitors
IQR: Interquartile range
LDL: Low-density lipoprotein
LSM: Liver stiffness measurement
MAFLD: Metabolic dysfunction-associated fatty liver disease
NAFLD: Non-alcoholic fatty liver disease
NASH: Non-alcoholic steatohepatitis
NFS: NAFLD fibrosis score
PI: Protease inhibitor
NRTI: Nucleoside reverse-transcriptase inhibitors
NNRTI: Non-nucleoside reverse-transcriptase inhibitors
PLWH: People living with HIV
TAF: Tenofovir alafenamide
TDF: Tenofovir disoproxil fumarate
NIT: Non-invasive test
VCTE: Vibration controlled transient elastography
