# Supplementary material for: Metabolic dysfunction-associated fatty liver disease in people living with HIV
Source: Sci Rep. 2023 Jun 6;13:9158. doi: 10.1038/s41598-023-32965-y (PMC10244325; doi:10.1038/s41598-023-32965-y)
Supplement: Supplementary file 1 — Supplementary Information. [file 41598_2023_32965_MOESM1_ESM.docx]

**Supplement**

**Supplementary Table 1 Comparison of HIV-related parameters between PLWH and non-MAFLD vs. MAFLD**

|  |  |  |  | **P** |
| --- | --- | --- | --- | --- |
| **Variable** | **Non-MAFLD** | **MAFLD** | **NAFLD** | **Non-MAFLD vs. MAFLD** |
| **N** | **207** | **75** | **76** |  |
| **HIV-related parameters** |  |  |  |  |
| RNA viral load n = 274 |  |  |  | 0961 |
| < 50 copies/ml | 126 (62.7) | 46 (63.0) | 48 (64.9) |  |
| > 50 copies/ml | 75 (37.3) | 27 (36.9) | 26 (35.1) |  |
| CD4 (cells/µl) n = 282 | 696 (515; 888.8) | 799 (533.5; 1019.3) | 802.5 (533.5; 1019.3) | 0.100 |
| > 500 CD4 cells/µl | 152 (73.4) | 55 (73.3) | 55 (72.4) | 0.987 |
| CDC stages |  |  |  | 0.657 |
| A | 59 (43.7) | 21 (42.0) | 23 (44.2) |  |
| B | 32 (23.7) | 15 (30.0) | 16 (30.8) |  |
| C | 44 (32.6) | 14 (28.0) | 13 (25.0) |  |
| NRTI (TAF vs. TDF) |  |  |  | 0.265 |
| TAF | 127 (83.6) | 52 (89.7) | 52 (88.1) |  |
| TDF | 25 (16.4) | 6 (10.3) | 7 (11.9) |  |
| INSTI | 134 (65.7) | 54 (73.9) | 52 (70.3) | 0.193 |
| DTG | 50 (24.2) | 18 (24.0) | 15 (19.7) | 0.979 |
| EVG | 41 (19.8) | 14 (18.6) | 14 (18.4) | 0.831 |
| BIC | 28 (13.5) | 17 (22.7) | 18 (23.7) | 0.064 |
| PI | 29 (14.1) | 10 (13.7) | 8 (10.7) | 0.925 |
| NRRTI | 47 (23) | 15 (20.5) | 17 (22.9) | 0.661 |

Data are expressed as numbers, median, percentage (%) or interquartile ranges (IQR 25th; 75th). P-values refer to the comparison between non-MAFLD vs. MAFLD. Boldface indicates statistical significance. A p-value < 0.05 was considered statistically significant.

**Supplementary Table 2 Comparison of HIV-related parameters between overlap MAFLD/NAFLD and MAFLD-only or NAFLD-only**

|  |  |  |  | **P** |  |  |
| --- | --- | --- | --- | --- | --- | --- |
| **Variables** | **Overlap MAFLD/NAFLD** | **MAFLD-only** | **NAFLD-only** | **Overlap vs. MAFLD-only** | **Overlap vs. NAFLD-only** | **MAFLD-only vs. NAFLD-only** |
| **N** | **65** | **10** | **11** |  |  |  |
| **HIV-related parameters** |  |  |  |  |  |  |
| RNA viral load |  |  |  | 0.832 | 0.554 | 0.537 |
| < 50 copies/ml | 40 (63.5) | 6 (60) | 8 (72.7) |  |  |  |
| > 50 copies/ml | 23 (36.5) | 4 (40) | 3 (27.3) |  |  |  |
| CD4 (cells/µl) | 802.5 (522.5; 1022.5) | 788 (593.5; 1029) | 786.5 (514; 982.3) | 0.935 | 0.981 | 0.880 |
| > 500 CD4 cells/µl | 47 (72.3) | 8 (80) | 8 (72.7) | 0.609 | 0.977 | 0.696 |
| CDC stages |  |  |  | 0.426 | 0.917 | 0.627 |
| A | 19 (43.2) | 2 (33.3) | 4 (50.0) |  |  |  |
| B | 14 (31.8) | 1 (16.7) | 2 (25.0) |  |  |  |
| C | 11 (25.0) | 3 (50.0) | 2 (25.0) |  |  |  |
| NRTI (TAF vs. TDF) |  |  |  | 0.715 | 0.217 | 0.605 |
| TAF | 46 (90.2) | 6 (90.0) | 6 (80.0) |  |  |  |
| TDF | 5 (9.8) | 1 (10.0) | 2 (20.0) |  |  |  |
| INSTI | 47 (74.6) | 7 (70.0) | 5 (45.4) | 0.758 | 0.051 | 0.256 |
| DTG | 14 (21.5) | 4 (40.0) | 1 (9.1) | 0.203 | 0.337 | 0.097 |
| EVG | 12 (18.5) | 2 (20.0) | 2 (18.2) | 0.907 | 0.982 | 0.916 |
| BIC | 16 (24.6) | 1 (10.0) | 2 (18.2) | 0.304 | 0.643 | 0.593 |
| PI | 7 (10.9) | 3 (30) | 1 (9.1) | 0.101 | 0.842 | 0.223 |
| NRRTI | 12 (19) | 3 (30) | 5 (45.4) | 0.426 | 0.055 | 0.466 |

Data are expressed as numbers, median, percentage (%) or interquartile ranges (IQR 25th; 75th). Boldface indicates statistical significance. A p-value < 0.05 was considered statistically significant.
